# Choroidal Neovascularization in Pediatric Patients: Analysis of Etiologic Factors, Clinical Characteristics and Treatment Outcomes

**DOI:** 10.3389/fmed.2021.735805

**Published:** 2021-11-29

**Authors:** Ting Zhang, You Wang, Wenjia Yan, Yafen Liu, Jinglin Lu, Limei Sun, Songshan Li, Li Huang, Zhaotian Zhang, Xiaoyan Ding

**Affiliations:** ^1^State Key Laboratory of Ophthalmology, Zhongshan Ophthalmic Center, Sun Yat-Sen University, Guangzhou, China; ^2^Guangdong Provincial Key Laboratory of Ophthalmology and Visual Science, Guangdong Provincial Clinical Research Center for Ocular Diseases, Guangzhou, China

**Keywords:** pediatric choroidal neovascularization, etiologic factor, congenital/developing abnormality, inflammatory retinochoroidopathy, anti-vascular endothelial growth factor

## Abstract

**Background and Objectives:** Choroidal neovascularization (CNV) is a common pathologic lesion that occurs in various chorioretinopathy, but very limited published data have reported in pediatric patients. This study aimed to investigate the etiologic factors, clinical features, and treatment outcomes of choroidal neovascularization (CNV) in children.

**Methods:** In this study, 33 eyes in 30 patients aged 18 years or younger with CNV were included. Comprehensive ophthalmic examination was performed in all the patients. The demographic profiles, laterality, visual acuity, optical coherence tomographic findings, fundus fluorescein angiographic findings, and the underlying pathology were analyzed. The types, locations, treatment outcomes, and recurrences of CNV were noted.

**Results:** The average age was 11.2 ± 4.6 (range, 1–18) years. Most CNVs affecting children were classic and type 2. The most common etiologic factors of CNV in pediatric patients were congenital/developing abnormalities (9/30, 30.0%) and inflammatory retinochoroidopathy (9/30, 30.0%), followed by idiopathic CNV (8/30, 26.7%). Subtype analysis showed that the etiologic factor was inflammatory retinochoroidopathy in children 12 years or older, whereas congenital/developing abnormalities were present in children younger than 12 years. Eyes with active CNVs required a mean of 1.40 ± 0.58 injections. No recurrence was observed during follow-up.

**Conclusions:** The etiologic factors of CNV in young Chinese patients were diverse, with congenital/developing abnormalities, inflammatory retinochoroidopathy and idiopathic CNV being the 3 most common ones. Eyes with active CNVs had good responses to antivascular endothelial growth factor treatment with low recurrence.

## Introduction

Choroidal neovascularization (CNV) is a common pathologic lesion that occurs in various chorioretinopathy. The most common cause of CNV is age-related macular degeneration, followed by pathologic myopia (1). However, in children and adolescents, the reasons related with CNV are diverse and the lesion have a severe impact on visual acuity and quality of life over patients' lifetime (2–4). Although the incidence of CNV is quite rare in children and adolescents, its impact in view of the number of blind years lived is tremendous (5).

In the pediatric population, CNV has been reported to be collected with myopia, infection, inflammation, congenital anomalies, retinal dystrophies, and may also be idiopathic (2). The management of CNV in the pediatric patient setting is challenging, and a number of options, such as observation, photodynamic therapy, laser photocoagulation and anti-vascular endothelial growth factor (anti-VEGF) treatment has been reported, variable visual outcomes has been observed (6–9). However, because of the lack of complaints and symptoms, early diagnosis and regular monitoring of CNV is difficult in young children.

So far, very limited published data have reported the etiologic factors, clinical characteristics, natural history, and treatment outcomes of CNV in pediatric patients, and the observed subjects were mostly Western (10). Hence, we performed this study to investigate the etiologic factors, clinical characteristics, and treatment outcomes of CNV in Chinese pediatric patients.

## Materials and Methods

This was a consecutive case series of patients aged 18 years or younger with CNV who were referred to Zhongshan Ophthalmic Center in Guangzhou, China, from January 2014 to September 2020. The study protocol was approved by the Institutional Review Board at Zhongshan Ophthalmic Center, Sun Yat-sen University, and was in accordance with the tenets of the Declaration of Helsinki. Informed consent was obtained from the parents of all the patients.

Complete ophthalmic examination was performed in children with CNV and their family members, including the visual acuity test, intraocular pressure test, slit-lamp biomicroscopy, optical coherence tomography (OCT), optical coherence tomography angiography (OCTA), fundus autofluorescence (FAF), fundus fluorescein angiography (FFA) and indocyanine green angiography (ICGA). The diagnosis of CNV was made on the basis of fundus findings, OCTA and FFA. Clinical data were collected, including age at presentation, gender, laterality, refractive errors, axial length, family history, and ocular findings in their family members.

With FFA and OCT, CNV lesions were classified as type 1 [within the sub-retinal pigment epithelium (RPE) space, typically corresponding to angiographically occult CNV], type 2 (within the subretinal space, typically corresponding to angiographically classic CNV), and type 3 (intraretinal retinal angiomatous proliferation) (11). CNVs were identified as active in case of any of the following findings: clinical evidence of exudate, presence of fluid or hemorrhage, leakage on FFA, and presence of sub- or intraretinal fluid on OCT. With or without treatment, the lesion was considered regressed if there was no hemorrhage clinically, no dye leakage on FFA, and no sub- or intraretinal fluid on OCT. CNV was considered stable if lesion characteristics and visual acuity (if available) remained unchanged for at least 6 months. We defined recurrence as the reappearance or worsening of lesion activity after complete regression or stabilization. The types of lesions, frequency of treatment, duration of follow-up, and recurrence rate of CNV were recorded. According to FFA and OCT, if CNV included the central fovea, the location of CNV was classified as subfoveal; if the margin of CNV was within 200 μm from the central fovea, it was classified as juxtafoveal (12). Peripapillary CNV (ppCNV) is defined as CNV located within 1 disc diameter of the margin of the optic nervehead (13).

The etiology of CNV was documented as follows. Simple high myopia was defined as a refractive error of −6 diopters (D) or worse, whereas pathologic myopia was defined as an refractive error of −6 D or worse, along with fundus changes such as diffuse/patchy chorioretinal atrophy, macular atrophy, lacquer cracks, or posterior staphyloma (14). Multifocal choroiditis was observed as chorioretinal lesions extending to the periphery and was associated with peripheral inflammation or panuveitis (15). The diagnosis of Best vitelliform macular dystrophy was confirmed by typical macular lesions and genetic testing. Other etiologies of CNV—such as optic disc hamartoma, ocular toxoplasmosis, optic disc drusen, and morning glory syndrome (MGS) —were defined on the basis of the presence of specific clinical and angiographic findings. If a case of CNV could not be attributed to any etiology, it was defined as idiopathic.

Statistical analyses were performed using SPSS Statistics for Windows (v23; IBM Corp, Armonk, NY, USA). Continuous variables were presented as mean (SD) after assessing for normality by inspecting histograms and were compared using the unpaired *t*-test. The chi-squared or Fisher exact test was used for categorical data. Statistical significance was defined as a *P*-value < 0.05.

## Results

### Characteristics of CNV in Children

A total of 30 pediatric patients (33 eyes) with a mean age of onset 11.2 ± 4.6 (range, 1–18) years were included in this study. The youngest patient was 1.8 years old. The number of males and females was 17 (56.7%) and 13 (43.3%), causing a male-to-female ratio of 1.3:1.0. The mean age of onset in males and females was 12.5 ± 3.9 and 10.1 ± 4.9 years, respectively, with no statistical differences (*P* = 0.39). Among the participants, 29 were Hans and 1 was Russian. The average duration of follow-up was 29.0 ± 13.8 months, ranging from 6 to 60 months. None of the patients had any systemic diseases. Unilateral presentation was the most common, which was found in 27/30 (90.0%) children. Bilateral CNVs were noted in 3/30 (10.0%) children. Best corrected visual acuity (BCVA) was present in 87.9% (29/33) eyes, and the mean BCVA was 0.95 ± 0.63 logMAR.

Among the 33 affected eyes with CNV, the majority (32/33, 97.0%) were with type 2 CNV, only 1/33 (3.0%) eye was with type 1, and no eye with type 3; 84.8% (28/33) CNVs were active and 15.2% (5/33) inactive; 84.8% (28/33) CNVs were located subfoveally, and 15.2% (5/33) CNVs were located in the peripapillary area (Table 2). All 5 eyes with ppCNV had congenital pathology: optic disc drusen (1 eye), morning glory syndrome (1 eye), congenital optic disc hamartoma (2 eyes), and retinitis pigmentosa (1 eye), and 80.0% (4/5) ppCNVs were active. The demographic profiles, characteristics of all affected eyes, and etiologic factors are presented in Tables 1, 2.

**Table 1:** Demographic profile and ocular associations.

**Features**	**Numbers**
Subjects (*n*)	30 (33 eyes)
**Presenting visual acuity**
**Age (years)**
Mean	11.2 ± 4.6
Male	12.5 ± 3.9
Female	10.1 ± 4.9
Median	11
Range	1–18
**Gender**
Male	17 (56.7%)
Female	13 (43.3%)
**Laterality**
Unilateral	27 (90.0%)
Bilateral	3 (10.0%)
**Ocular associations**
Congenital/developing abnormalities	9 (30.0%, 10 eyes)
Best vitelliform macular dystrophy	3 (10.0%, 4 eyes)
Retinitis pigmentosa	2 (6.7%, 2 eyes)
Optic disc drusen	1 (3.3% 1 eye)
Morning glory disc anomaly	1 (3.3% 1 eye)
Optic disc hamartoma	2 (6.7%, 2 eyes)
Inflammatory retinochoroidopathy	9 (30.0%, 10 eyes)
Ocular toxoplasma retinochoroiditis	5 (16.7%, 6 eyes)
Ocular toxocariasis retinochoroiditis	1 (3.3%, 1 eye)
Multifocal choroiditis	3 (10.0%, 3 eyes)
Idiopathic	8 (26.7%, 8 eyes)
High myopia	4 (13.3%, 5 eyes)

**Table 2:** Characteristics of CNVM.

**Feature**	***n* (%)**
**Location (*****n*** **=** **33)**	
Subfoveal	28 (84.8)
Peripapillary	5 (15.2)
**Activity at presentation (*****n*** **=** **33)**	
Active	28 (84.8)
Inactive	5 (15.2)
**Pattern of leakage in active CNVMs where FFA was available (*****n*** **=** **28)**	
Classic	27 (96.4)
Occult	1 (3.6)
**Types of CNVMs where OCT was available (*****n*** **=** **33)**	
Type 1	1 (3.0)
Type 2	32 (97.0)

### Analysis of Etiologic Factors Associated With CNV in Children

**Figure 1:**
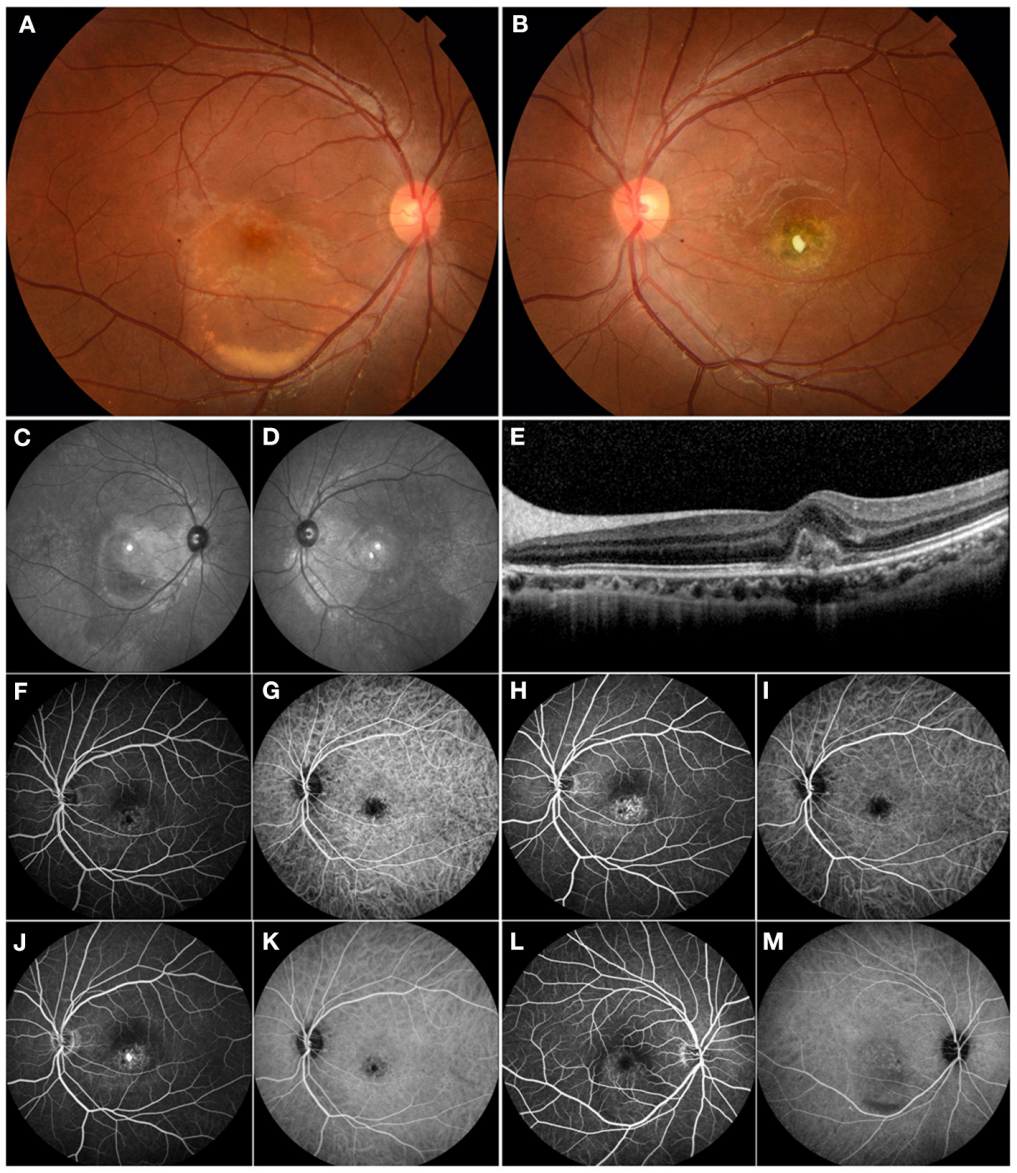
Multimodal imaging of a 17-year-old male who presented with choroidal neovascularization (CNV) related to Best vitelliform macular dystrophy. His family history was unremarkable. His BCVA was 0.1 logMAR in the right eye and 1.1 logMAR in the left eye. **(A)** Fundus examination of the right eye revealed a color photo: a vitelliform lesion in the submacular area indicating Best disease. **(B)** The left eye showed a yellow-white fibrotic membrane—an area of atrophy and pigmentation. **(C,D)** The red-free images showed macular hyperautofluorescence in both eyes. **(E)** OCT revealed the typical subfoveal hyperreflective at the level of the RPE. **(F,H,J)** FFA demonstrated mild hyperfluorescence in the macular area and frank hyperfluorescence in the late phase consistent with subfoveal CNV in the left eye. **(G,I,K)** ICGA showed hypofluorescence in the macular area consistent with the lesion. **(L)** FFA demonstrated mild hyperfluorescence in the macular area secondary to a vitelliform lesion in the right eye. **(M)** ICGA showed mild hypofluorescence secondary to a vitelliform lesion in the right eye. The patient underwent intravitreal ranibizumab treatment for his left eye. However, he did not notice any significant visual improvement.

**Figure 2:**
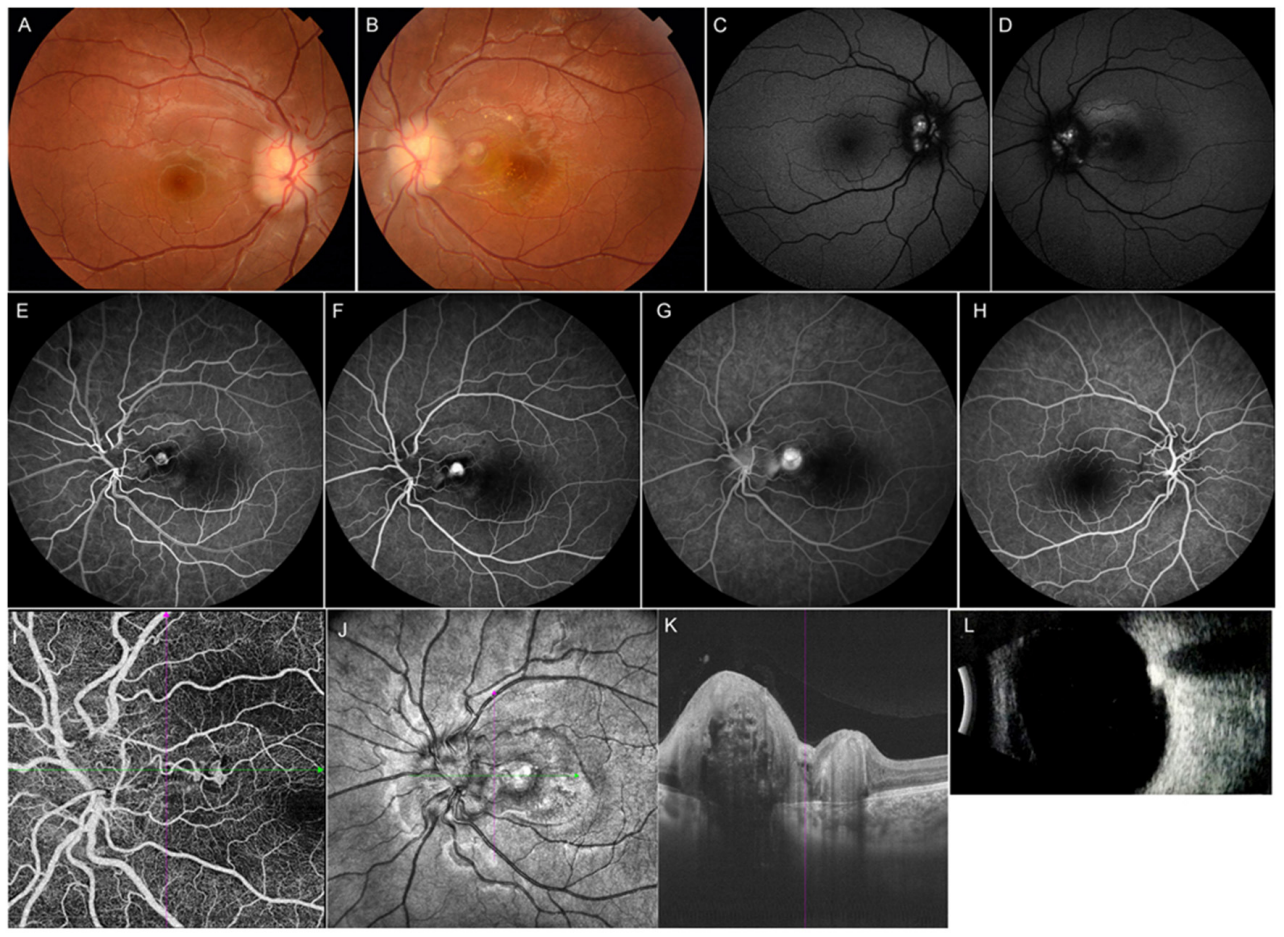
Multimodal imaging of an 11-year-old female with optic disc drusen. Her BCVA was 0.8 logMAR in the left eye and 0.1 logMAR in the right eye. Her ocular and systemic history was unremarkable. **(A,B)** Fundus photograph showed elevated optic discs with blurred margins in both eyes. **(C,D)** Mild hyperautofluorescence was observed around the disc bilaterally. **(E–G)** FFA showed a lesion located in the peripapillary area with hyperfluorescence in the early phase and leakage in the late phase in the left eye. **(H)** FFA image of the right eye. **(I,J)** OCT angiography clearly showed CNV located in the peripapillary area. **(K)** OCT image of the left eye revealed subretinal hyperreflectivity. **(L)** Ultrasound B-scan showed a strong signal in front of the optic disc. The girl underwent intravitreal ranibizumab treatment, and her BCVA improved to 0.1 logMAR at final follow-up.

**Figure 3:**
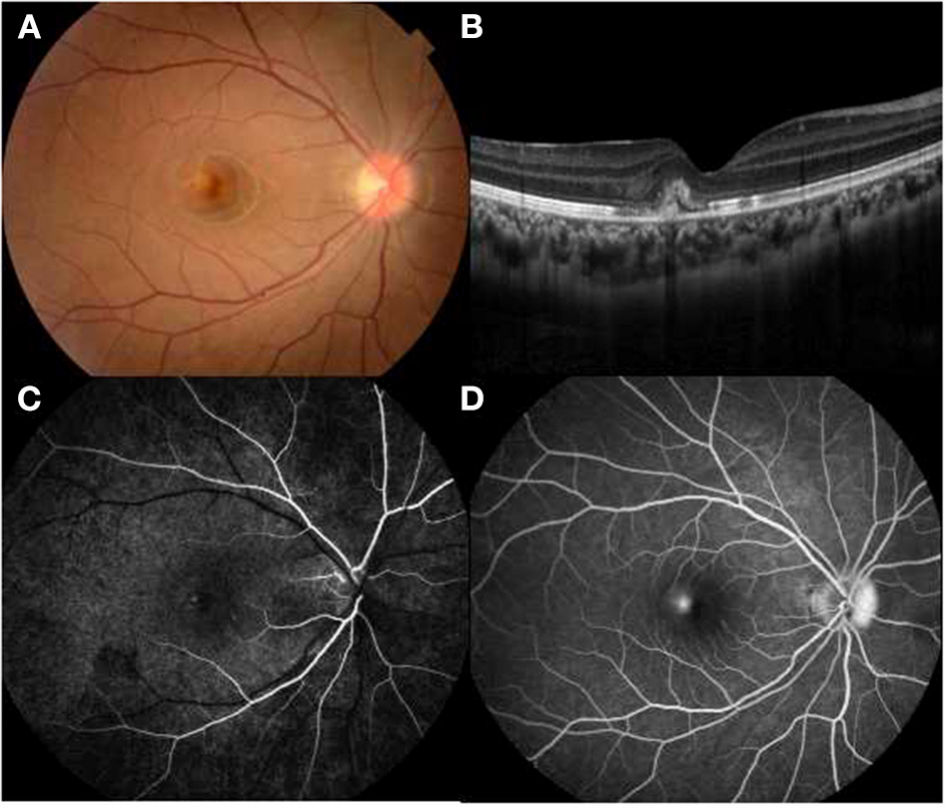
Multimodal imaging of a 14-year-old female with idiopathic choroidal neovascularization. Her BCVA was 0.9 logMAR in the right eye and 0.1 logMAR in the left eye. **(A)** A parafoveal yellowish lesion was noted on funduscopy. **(B)** OCT showed a hyperreflective area located in subretinal space with arounding serous retinal detachment. **(C,D)** Hyperfluorescence in the early phase and leakage in the late phase FFA revealed an active lesion parafoveally.

**Figure 4:**
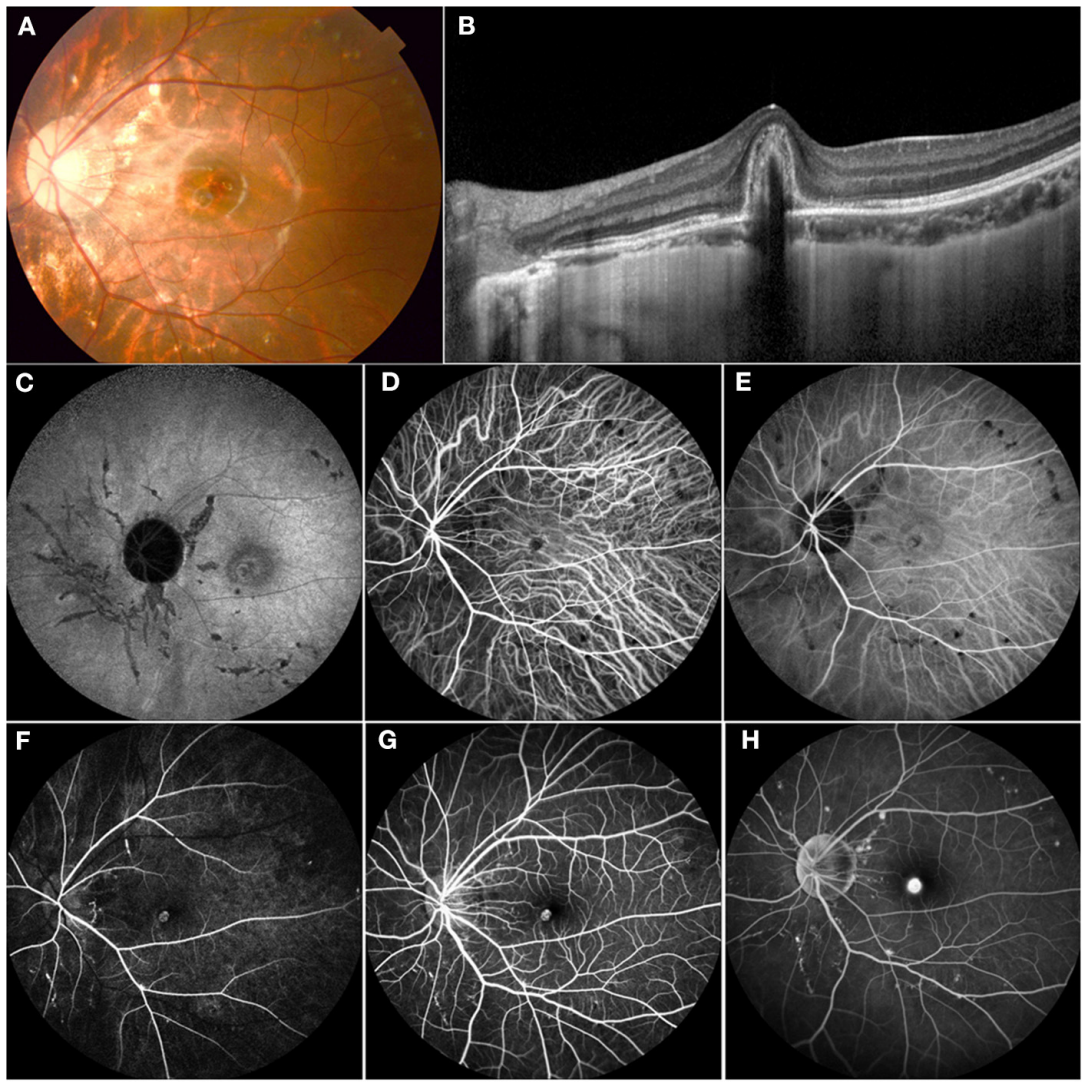
Multimodal imaging of an 11-year-old female who presented with decreasing vision in her left eye. Her BCVA was 0.3 logMAR with a refraction of −6.00 – 1.50*5 in the left eye and 0.9 logMAR with a refraction of −5.5 – 1.25*177 in the right eye. **(A)** Color fundus demonstrated mottled fundus, lacquer cracks, and myopia conus. **(B)** OCT showed a subretinal high-refractive material with fuzzy margins and the absence of the inner segment/outer segment junction. **(C)** FAF showed hypofluorescence secondary to mechanical linear breaks in the elastic layer of the Bruch membrane. **(D,E)** ICGA showed an abnormal vascular network and hypofluorescence corresponding to the areas of LC. **(F–H)** FFA demonstrated hyperfluorescence in the early phase and leakage in the late phase of CNV and a hyperfluorescence linear area consistent with LC. This patient received 1 intravitreal injection of ranibizumab, and the vision was stable at 0.3 at follow-up.

A wide range of etiologic factors associated with CNV were identified in this study. Diverse congenital retinal or optic disc anomalies were seen in 10 eyes in 9 children, including Best vitelliform macular dystrophy (4 eyes in 3 children) (Figure 1), retinitis pigmentosa (2 eyes in 2 children), optic disc drusen (1 eye in 1 child) (Figure 2), morning glory disc anomaly (1 eye in 1 child), and optic disc hamartoma (2 eyes in 2 children). Inflammatory retinochoroidopathy was observed in 10 eyes in 9 children, including toxoplasma chorioretinitis (6 eyes in 5 children) (Figure 3), multifocal choroiditis (3 eyes in 3 children), and ocular toxocariasis (1 eye in 1 child). Simple high myopia was identified in 4 eyes in 3 children, and 1 eye with pathologic myopia with fundus changes was found (Figure 4). In addition, idiopathic CNV was defined in 8 eyes in 8 children. Overall, within these associated factors, congenital retinal or optic disc anomalies (9/30, 30.0%) and inflammatory retinochoroidopathy (9/30, 30.0%) are the most common etiologic factors, followed by idiopathic CNV (8/30, 26.7%). Details of all affected eyes are presented in Table 1.

Subgroup analysis based on the age of onset showed the most common etiologic factor of CNV in patients 12 years or older was inflammatory chorioretinopathy (5/13, 38.5%), followed by high myopia (4/13, 30.7%), idiopathic CNV (3/13, 23.1%), and congenital/developmental anomalies (1/12, 7.7%). However, congenital/developmental anomalies were the major etiologic factor in patients younger than 12 years (8/17, 47.1%), followed by idiopathic CNV (5/17, 29.4%) and inflammatory chorioretinopathy (4/17, 23.5%). A statistically significant difference was found in etiology between patients 12 years or older and those younger than 12 years (χ = 9.3, *P* = 0.02) (Table 3).

**Table 3:** Distribution of etiology by age range for patients with choroidal neovascular membrane.

**Etiologic factors**	***N* (%)**	**Subgroup analysis**	
		**0–11 years, *N***	**12–18 years, *N***
Congenital retina and optic disc anomalies	9 (30.0%)	8 (47.1%)	1 (7.7%)
Best vitelliform macular dystrophy	3	2	1
Retinitis pigmentosa	2	2	0
Optic disc drusen	1	1	0
Morning glory disc anomaly	1	1	0
Optic disc hamartoma	2	2	0
Inflammatory retinochoroidopathy	9 (30.0%)	4 (23.5%)	5 (38.5%)
Ocular toxoplasma retinochoroiditis	5	3	2
Ocular toxocariasis retinochoroiditis	1	1	0
Multifocal choroiditis	3	0	3
Idiopathic	8 (26.7%)	5 (29.4%)	3 (23.1%)
High myopia	4 (13.3%)	0	4 (30.7%)
Total	30	17	13

Among these 30 children, three cases presented with bilateral CNVs, including Best disease (1), high myopia (1), and toxoplasma (1). The patient with Best disease has active stage III lesion in both eyes at the time of diagnosis. The bilateral CNVs in high myopia were active. However, the bilateral CNVs secondary to toxoplasma were inactive and this child was left to observe without any treatment.

### Anti-VEGF Treatment and Outcomes of CNV in Pediatric Patients

According to the reported studies and our previous experience with myopic CNV in adults, where the majority belonged to type 2 CNV, one anti-VEGF injection followed by a pro re nata (1 + PRN) regimen was found to be quite effective (16). Thus, the 1+ PRN treatment modality was used in 25 eyes in 23 children with active CNV lesions in this study. The eye with active CNV due to toxocariasis uveitis was treated with pars plana vitrectomy combined with oral anti-inflammatory glucocorticoid. Two eyes in 2 children did not receive treatment because of rejection from their guardians. Five eyes with inactive CNVs were left to observe, including 2 eyes in 1 patient with toxoplasma. Standard dose of anti-VEGF (aflibercept 2 mg and ranibizumab 0.5 mg) was used in this study.

The outcome of anti-VEGF treatment for CNVs in 25 eyes (23 children) is summarized in Table 4. BCVA was available in 23/25 eyes. The average baseline BCVA before anti-VEGF treatment was 0.96 ± 0.49 logMAR; it increased to 0.85 ± 0.44 logMAR 3 months after the treatment and stabilized at 0.85 ± 0.42 logMAR at final follow-up. No significant difference was noted in comparing BCVA pre- and post-treatment (*P* > 0.05). A slight improvement in BCVA was seen in 12 (12/23, 52.1%) eyes at follow-up, including 6 (6/6, 100%), 1 (1/7, 14.3%),1 (1/5, 20%) and 4 (4/5, 80%) eyes with idiopathic CNV, congenital retina and optic disc anomalies, high myopia and inflammatory retinochoroidopathy, respectively. No significant changes were observed in 11 (11/23, 47.9%) eyes at follow-up, including 0 (0/6, 0%), 6 (6/7, 85.7%), 4 (4/5, 80%), and 1 (1/5, 20%) eyes with idiopathic CNV, congenital retina and optic disc anomalies, high myopia and inflammatory retinochoroidopathy, respectively. The average frequency of initial anti-VEGF treatment was 1.40 ± 0.58 injections. Among the eyes that received 1+ PRN intravitreal anti-VEGF treatment as the loading dose, CNVs in 16/25 (64.0%) eyes in 14 children were stabilized with 1 injection only, whereas 8/25 (32.0%) eyes received a second injection during the loading stage at treatment initiation, and 1 eye received a third injection. No recurrence occurred during the study period. CNVs in 25/25 (100.0%) eyes were stabilized at final follow-up. There were no significant ocular or systemic complications in these children.

**Table 4:** Treatment profile.

**Treatment types (*n* = 25)**	***N*/$\bar{\text{x}}$±*s***
Ranibizumab	17
Conbercept	5
Aflibercept	3
**Average frequency**	1.40 ± 0.58
1 injection	16
2 injections	8
3 injections	1
**BCVA (23 eyes, logMAR)**
Baseline	0.96 ± 0.49
Post-treatment	0.85 ± 0.443
Final visit	0.85 ± 0.42
**Outcome of BCVA (*****n*** **=** **23)**
Improvement	12
Stability	11

## Discussion

CNV is quite a rare, but sight-threatening disease affecting children and adolescents. The first and only available population-based incidence of CNV was reported recently by Moosajee et al. from the United Kingdom in those aged 16 years or younger, with an annual incidence of 0.21 per 100 000 (2). To date, there are no other detailed data on the prevalence of CNV in the pediatric population, and most knowledge of this topic comes from case series or single case reports. Because of the low incidence, only a few series of CNVs in children have been reported. To the best of our knowledge, this is the first study to report the etiologic factors, clinical characteristics, and treatment outcomes of CNV in a series of Chinese pediatric population younger than 18 years.

### Etiologic Factors and Clinical Characteristics

In this study including 33 eyes of 30 pediatric patients with CNV, at least 1 contributing etiology could be identified in 25/33 (75.8%) eyes of the patients. A study by Tapas et al. demonstrated that retinal dystrophies had the leading ocular correlation with CNV in pediatric patients younger than 18 years (10). In our series, identifiable ocular association could not be found in only 8/30 (26.7%) patients, whereas congenital/developing anomalies and inflammatory retinochoroidopathy were noted to be the major etiologic factors of CNV in children. Among them, Best vitelliform macular dystrophy was the most common reason, which is in line with the study of Tapas et al. (10). Furthermore, one of the strengths of our study is the findings concerning the distribution of etiology. Subgroup analysis based on the age of the patients at the onset of CNV showed that the most common etiology of CNV in patients younger than 12 years was congenital/developmental anomalies (8/17, 47.1%), whereas inflammatory retinochoroidopathy was the major etiologic factor in patients older than 12 years (5/11, 38.5%).

Moreover, in previous studies on CNV in the pediatric population, high myopia had a low incidence, especially in studies conducted in the West (5). However, our data showed that high myopia (4/11, 30.7%) plays an important role in Chinese patients 12 years or older with CNV, which was probably because myopia is more prevalent in Eastern Asians (17). Interestingly, in adults, subretinal CNV generally develops in an eye with diffuse or patchy macular atrophy and lacquer cracks, which was identified as pathologic myopia. CNVs are usually present at the edges of lacquer cracks, atrophy plaque, or steep staphylomatous area (18, 19), whereas in pediatric patients in our study, they were observed in 1 pathologic myopic eye with lacquer cracks and 3 highly myopic eyes without evident myopic fundus changes at the posterior pole, which was identified as simple high myopia. All CNVs due to simple high myopia were seen in teenagers, ranging from 14 to 18. The underlying pathogenesis is still elusive. We suggest that the contribution of dramatic elongation of the globe during adolescence is considered, which may produce biomechanical stretching of the retina, RPE, and choroid with a straightening and thinning of retinal vessels with reduction of retinal vascular flow and a diminished density of the retinal capillary network and choriocapillaris (20).

Because of the rarity of ppCNV in the pediatric population, the natural history, prognosis, and treatment strategy are not clear so far. In the current series, all 5 ppCNVs occurred in patients younger than 12 years who had a preexisting ocular pathology, including optic nervehead drusen, optic disc hamartoma, morning glory disc anomaly, and retinitis pigmentosa. In our series, 4/5 (80%) ppCNVs were active. The natural course of untreated ppCNVs has been reported to be variable by ranging from spontaneous involution to fulminant enlargement toward the fovea, which is vision threating (21). Thus, in our series, all the active lesions, although some of them were not affecting the vision, were treated with anti-VEGF therapy. CNVs responded well with an average of 1.25 injections. Non-active CNVs associated with MGS were left to observe and kept stable within the 36 months of follow-up.

### Treatments and Outcomes

Children with CNVs seem to respond well to anti-VEGF treatment. Kozak et al. analyzed the data of 45 eyes in 39 children with intravitreal bevacizumab or ranibizumab for CNV over a mean follow-up period of 12.8 months. 2.2 injections per eye was required for treatment. An improvement in BCVA of 3 lines was seen in 22 (49.0%) eyes, and only 1 eye had worsened vision after treatment (7). In our study, it was found that an average of 1.40 ± 0.58 anti-VEGF injections were needed for the regression or stabilization of CNV membrane (CNVM), which is significantly lower than what is commonly seen with CNVM in adults, even less than that reported in a study by Kozak et al. (7, 16). Injections on a 1+PRN basis demonstrated similar results as monthly injections. The need for retreatments is definitely much less than that for adults in prior reports (16). In our series, it was usually between 1 and 3 in only 36% of children. However, the number of patients in this study was too small to draw any clear conclusions regarding which options performs better for post-treatment recurrence. However, visual improvement is poor in CNVs in the pediatric population after the stabilization, or even regression, of CNV lesions. In our series, 12/23 (52.1%) patients showed a slight improvement, whereas 11/23 (47.9%) did not show any significant improvement in visual acuity during follow-up. Similarly, 90% of the patients in a case series of Goshorn et al. with initial visual acuity of <1.0 logMAR remained unchanged (22). The improvement in BCVA was seen in 6 (6/6, 100%) eyes with idiopathic CNV and 4 (4/5, 80%) eyes with inflammatory retinochoroidopathy, respectively. The results showed better treatment effects in idiopathic CNV and inflammatory retinochoroidopathy.

CNV in children has a more favorable prognosis than in adults with AMD, even if left for observation. In our cohort, 2 eyes with active CNV in 2 patients were left to observe because of rejection from their guardians, including 1 eye with retinitis pigmentosa and 1 with ocular toxoplasma retinochoroiditis. Both eyes had a spontaneous regression of CNV, although visual acuity did not show any improvement. Five eyes with inactive CNV in 4 patients still remained inactive, and no improvement in BCVA was observed during follow-up. Goshorn et al. reported spontaneous regression of CNV in 11/19 (58.0%) untreated eyes in children and 9 patients obtained visual acuity better than or equal to 20/50 (22). Rishi et al. reported spontaneous involution of CNV in 15/17 untreated eyes and suggested the less need for treatment was related to the better health of RPE pump in children (3). RPE play an important role by mixing with fibrocytes, collagen, vascular endothelium and lymphocytes to form the CNV. In the late stage of CNV, the RPE proliferated to enclose CNV and caused its regression (23). Observation of CNVs in children might be a reasonable option; but, so far, it is difficult to assess which CNV would regress or progress. Moreover, Rishi et al. noted that visual outcome in eyes with treated CNV was better than in those with spontaneously regressed CNV (3). Thus, according to the effectiveness and favorable prognosis with limited anti-VEGF treatment, we suggest timely treatment is considered for CNV in the pediatric population.

There are several limitations of this study that need to be considered. First, as CNV is rare in the pediatric population, the number of included patients was limited. Second, because of the young age of the patients in this case series, some details—such as visual acuity, refractive status, clinical presentation, and OCT findings—were lacking. Third, referral biases might be existed as our hospital is a tertiary referral institute for pediatric retinal diseases. Fourth, the non-compliance of OCTA in young cases limited the consequent analysis of the microstructure modeling of CNV. Therefore, a prospective multicenter clinical study with more detailed objective information may be warranted. However, this study—with a relatively large sample size—was the first to reveal that the etiologic factors of CNV in the Chinese pediatric population varied significantly from those in adults.

In conclusion, we analyzed the etiologic factors, clinical features, and treatment outcomes of CNV in the pediatric population. Most of the CNVs were found to be classic on FFA and type 2 on OCT and had a subfoveal location. In our study, according to age, congenital/developmental abnormalities were the major etiologic factor in patients younger than 12 years, whereas inflammatory retinochoroidopathy was the most common reason in those older than 12 years. The high prevalence of inflammatory CNV in pediatric and young teenage patients catches the attention of ophthalmologists. Intravitreal anti-VEGF treatment on a PRN basis after the first injection was demonstrated to be effective. With a long follow-up, it has been shown that children with CNVs respond well to anti-VEGF treatment. In summary, CNV in the pediatric population in our study differed from that in the adult population according to etiology, angiographic characteristics, and treatment response.

## Data Availability Statement

The raw data supporting the conclusions of this article will be made available by the authors, without undue reservation.

## Ethics Statement

The studies involving human participants were reviewed and approved by the Institutional Review Board at Zhongshan Ophthalmic Center, Sun Yat-sen University. Written informed consent to participate in this study was provided by the participants' legal guardian/next of kin.

## Author Contributions

TZ, YW, and XD conceptualized and designed the study, drafted the initial manuscript, and reviewed and revised the manuscript. WY, YL, JL, and LS designed the data collection instruments, collected data, and carried out the initial analyses. SL, LH, and ZZ conceptualized and designed the study, coordinated and supervised data collection, and critically reviewed the manuscript for important intellectual content. All authors approved the final manuscript as submitted and agree to be accountable for all aspects of the work.

## Funding

This study is supported in part by grants from the Fundamental Research Funds of State Key Laboratory of Ophthalmology, research funds of Sun Yat-sen University (15ykjxc22d; Guangzhou, Guangdong, China), Science and Technology Program Guangzhou, China (201803010031; Guangzhou, Guangdong, China), the National Natural Science Foundation of China (no. 81900896), Science and Technology Program Guangzhou, China (202102010430010067; Guangzhou, Guangdong, China). The sponsors and funding organizations had no role in the design or conduct of this research.

## Conflict of Interest

The authors declare that the research was conducted in the absence of any commercial or financial relationships that could be construed as a potential conflict of interest.

## Publisher's Note

All claims expressed in this article are solely those of the authors and do not necessarily represent those of their affiliated organizations, or those of the publisher, the editors and the reviewers. Any product that may be evaluated in this article, or claim that may be made by its manufacturer, is not guaranteed or endorsed by the publisher.
